# Relationship between serum uric acid level and mild cognitive impairment in Chinese community elderly

**DOI:** 10.1186/s12883-017-0929-8

**Published:** 2017-08-01

**Authors:** Miao Liu, Jianhua Wang, Jing Zeng, Yao He

**Affiliations:** 1 Institute of Geriatrics, Chinese PLA General Hospital, 28 Fuxing Road, Beijing, 100853 China; 2 Beijing Key Laboratory of Aging and Geriatrics, Beijing, China; 3 State Key Laboratory of Kidney Disease, Chinese PLA General Hospital, 28 Fuxing Road, Beijing, 100853 China

**Keywords:** Chinese, Elderly, Epidemiology, Mild cognitive impairment, Uric acid

## Abstract

**Background:**

To evaluate the association between SUA levels within a normal to high range and the risk of mild cognitive impairment (MCI) among community elderly.

**Methods:**

The present study was based on 2102 community elderly from a cross-sectional study conducted in a representative urban area of Beijing between 2009 and 2010. The mean age were 71.2 ± 6.6 years old, 59.7% were female. Mini-Mental State Examination (MMSE) was used to assess cognitive function by trained neurology doctors.

**Results:**

The prevalence of hyperuricemia and MCI was 16.7% and 15.9% respectively. With the increase of SUA levels, the prevalence of MCI showed a strong decreasing linear trend. Multiple logistic regression analysis showed ORs for MCI were 1.01(95% CI: 0.69–1.48), 1.50(95% CI: 0.85–2.64), 1.65(95% CI: 1.12–2.43) and 1.53(95% CI: 1.00–2.33), 1.84(95% CI: 1.27–2.90), 1.92(95% CI: 1.02–3.35) for the second, third and highest quarters among men and women respectively (with the lowest quartile as the reference).

**Conclusions:**

Higher SUA levels, when in the normal range, were positively associated with cognitive function among Chinese community elderly, but this association was not robust among participants with hyperuricemia.

**Electronic supplementary material:**

The online version of this article (doi:10.1186/s12883-017-0929-8) contains supplementary material, which is available to authorized users.

## Background

With the acceleration in the population aging, dementia has become one of the most important health problems over the world [1]. Studies have estimated that the total number of population affected by dementia is about 46.8 million globally, and the number keeps increasing [2, 3]. According to WHO’ report, the patients affected by dementia will double every 20 years, and most of them live in developing countries [4]. In China, dementia is also a serious problem along with the large elder population. It was estimated people with dementia in China increased from 3.68 million in 1990 to 9.19 million in 2010 [5]. On the other hand, the disease burden of dementia is huge. It will not only cause disability, reduce life quality of patients, and also causes burdens to caregivers and take up health and social care resources [6–8]. Hence, it’s of critical importance to carry out early prevention and control of dementia.

Several studies have focused on serum uric acid (SUA) level and cognitive function [9–15]. Like the old saying, gout unlike any other disease, kills more wise men than simple, which means that gout patients have higher SUA levels and also higher intelligence quotient (including cognitive function) [16].Although a number of studies have revealed the relationship, the conclusion remains controversial. Some studies found that higher SUA level was beneficial to cognitive function and prevent cognitive impairment [12, 13]. But other studies also came to the opposite conclusion; SUA was a risk factor for cognitive function, like for cardiovascular disease [10]. Also, most studies only included the normal SUA levels, but missed the very high SUA level [17–19]. Thus, SUA levels within a normal range have not been fully studied with cognitive function. Therefore, we examined the association of SUA level in the normal range and also in the hyperuricemia range for related risk of MCI in an urban community elderly population of Beijing, China.

## Methods

### Study population

The sampling methods were reported in our previous study [20]. In brief, this was a population-based cross-sectional survey conducted in a metropolitan area representative of urban community in Beijing, China. A two-stage stratified clustering sampling method was used. First, 9 residents were randomly selected from the total 94 residents in Wanshoulu district. Second, all the individuals aged ≥60 years that had lived in the selected residence for more than 1 year were recruited. A total of 2162 residents aged 60–95 years were selected and invited for screening. Besides the 60 elderly with incomplete data, the left 2102 residents (848 men, 1254 women) completed the survey, and the participants accounted for about 10% of total elderly in Wanshoulu community.

All the nurses and doctors who took part in the field survey had strict training. Face-face interview was carried out, all the detail health condition of the participants were asked using a standardized questionnaire, including demographic factors, medical history, family history, and lifestyles. Physical examinations was carried out by specially trained nurses, including height, weight, waist circumstance (WC). We also calculated body mass index (BMI). Two blood pressures were measured using sphygmomanometer in a sitting position after 30 min’ rest. Fasting blood specimens of all participants were obtained for standard tests of glucose, blood lipids and SUA level.

### Definitions

Hyperuricemia was defined positive if SUA level was ≥7.0 mg/dL (417 μmol/L) in men or ≥6.0 mg/dL (357 μmol/L) in women, or positively diagnosed [21]. SUA levels of participants with and without hyperuricemia were categorized into 4 levels using the quartiles (P_25_, P_50_ and P_75_) as cut-off values by gender.

We used the Mini-Mental State Examination (MMSE) to assess cognitive function by trained neurology doctors. The MMSE scale was proved to be feasible among Chinese for dementia screening [22]. MCI was defined according the guideline, which is as follows: (1) for illiteracy participants, MMSE score less than a score of 17; (2) for participants with 1–6 education years, MMSE score less than a score of 20; (3) for participants with more than 7 education years, MMSE score less than a score of 24 [23, 24].

Prevalence of cerebral vascular disease and kidney disease was defined according to previous medical diagnosis by hospitals of second level and above. Current smoking was defined as those who currently smoked at least one cigarette daily; current alcohol drinking was defined as drinking alcohol at least once weekly for the past year. Physical activity was divided into two categories: ≥0.5 h/day or <0.5 h/day.

### Statistical analysis

For the basic characteristics, x̄ ± s were used for continuous variables like age, WC, BMI, blood pressure, blood lipid, glycaemia and SUA level. *n* (%) were used for categorical variables like gender(male or female), education level (less than 6 years, or more than6 years), marriage status (married or unmarried), current smoking (yes or no), current drinking(yes or no), physical activity ≥ 0.5 h/day (yes or no), family history (yes or no). T test and Chi-square test were used to examine differences in continuous and categorical variables. Linear multiple regression and logistic multivariable regression was used to estimate the βand odds ratio (OR) of gender-specific SUA level with cognitive function. All analyses were conducted using SPSS (19.0, No. of Serial: 5,076,595).

## Results

### Clinical features of participants with and without hyperuricemia

The mean SUA level was 5.3 ± 1.4 mg/dL (range, 1.4–11.3 mg/dL), men had a higher SUA level than women (5.8 ± 1.3 vs 5.2 ± 1.4, *p* < 0.001). The general characteristics according to gender and hyperuricemia were shown in Table 1. The mean age were 71.2 ± 6.6 years old, 59.7% were female. The education level was high, 72.4% of all the participants had at least gone to junior middle school. Participants with hyperuricemia had older age, greater WC, higher BMI, higher TG, level and lower HDL-C levels. However, there were no significant differences in blood pressure and FPG. In women, besides these differences, we also observed higher levels of 2hPG in participants with hyperuricemia than those without. The percentage of participants who were married, better educated, current smoking and drinking did not differ with hyperuricemia. For MMSE score, participants with hyperuricemia had higher MMSE score in men (27.7 ± 2.6 vs 27.0 ± 3.8, *p* = 0.014) but not in women (27.0 ± 3.5 vs 26.7 ± 3.8, *p* = 0.057).

**Table 1:** General characteristics of the participants by hyperuricemia

Characteristic	Male (*n* = 848)				Female (*n* = 1254)				Total (*n* = 2102)
	hyperuricemia (*n* = 148)	No hyperuricemia (*n* = 700)	*P*	subtotal	hyperuricemia (*n* = 204)	No hyperuricemia (*n* = 1050)	*P*	subtotal	
*x̄ ± s*									
Age (yrs)	73.5 ± 6.9	72.1 ± 6.8	0.028	72.3 ± 6.9	71.8 ± 6.0	70.1 ± 6.3	0.001	70.4 ± 6.2	71.2 ± 6.6
WC(cm)	94.0 ± 8.4	90.6 ± 8.4	<0.001	91.2 ± 8.5	90.3 ± 9.5	85.6 ± 8.6	<0.001	86.4 ± 8.9	88.3 ± 9.1
BMI(kg/m2)	25.7 ± 3.0	24.8 ± 3.1	0.002	24.9 ± 3.1	26.4 ± 3.8	24.8 ± 3.5	<0.001	25.0 ± 3.6	25.0 ± 3.4
SBP(mm Hg)	136.8 ± 17.5	136.6 ± 18.0	0.927	136.7 ± 17.9	141.9 ± 20.4	140.2 ± 20.9	0.285	140.5 ± 20.8	139.0 ± 19.8
DBP(mmHg)	77.4 ± 9.4	78.6 ± 9.7	0.191	78.4 ± 9.7	75.9 ± 10.4	76.8 ± 9.9	0.273	76.6 ± 10.0	77.3 ± 9.9
TC(mmol/L)	5.0 ± 1.1	4.9 ± 0.9	0.131	4.9 ± 0.9	5.5 ± 1.0	5.5 ± 1.0	0.550	5.5 ± 1.0	5.3 ± 1.0
TG(mmol/L)	1.8 ± 1.0	1.5 ± 0.8	0.001	1.5 ± 0.9	2.1 ± 1.0	1.7 ± 0.9	<0.001	1.8 ± 0.9	1.7 ± 0.9
HDL-C(mmol/L)	1.2 ± 0.3	1.3 ± 0.4	0.016	1.3 ± 0.4	1.4 ± 0.4	1.5 ± 0.4	<0.001	1.5 ± 0.4	1.4 ± 0.4
LDL-C(mmol/L)	3.1 ± 0.9	3.0 ± 0.8	0.338	3.1 ± 0.8	3.4 ± 0.8	3.4 ± 0.9	0.506	3.4 ± 0.9	3.3 ± 0.8
FPG(mmol/L)	6.0 ± 1.3	6.1 ± 1.6	0.428	6.1 ± 1.5	6.1 ± 1.3	6.1 ± 1.8	0.704	6.1 ± 1.8	6.1 ± 1.7
2hPG(mmol/L)	8.1 ± 2.6	8.2 ± 3.0	0.707	8.2 ± 3.0	9.2 ± 3.3	8.2 ± 3.3	<0.001	8.4 ± 3.3	8.3 ± 3.2
MMSE score	27.7 ± 2.6	27.0 ± 3.8	0.014	27.1 ± 3.6	27.0 ± 3.5	26.7 ± 3.8	0.057	26.8 ± 3.7	26.9 ± 3.7
*n* (%)									
Education ≥ 7 yrs	124(83.8)	576(82.3)	0.663	700(82.5)	142(69.6)	680(64.8)	0.183	822(65.6)	1522(72.4)
Married	131(88.5)	650(92.9)	0.075	781(92.1)	157(77.0)	836(79.6)	0.392	993(79.2)	1774(84.4)
Current smoking	27(18.2)	153(21.9)	0.329	180(21.2)	7(3.4)	44(4.2)	0.615	51(4.1)	231(11.1)
Current alcohol drinking	58(39.2)	267(38.1)	0.812	325(38.3)	14(6.9)	86(8.2)	0.522	100(8.0)	425(20.2)
Physical activity (≥0.5 h/day)	88(59.5)	422(60.3)	0.852	510(60.1)	121(59.3)	603(57.4)	0.618	724(57.7)	1234(58.7)
Family history of dementia	10(6.8)	32(4.6)	0.266	42(5.1)	10(4.9)	50(4.8)	0.932	60(4.8)	102(4.9)
Cerebral vascular disease	28(18.9)	90(12.9)	0.053	118(13.9)	25(12.3)	124(11.8)	0.857	149(11.9)	267(12.7)
Kidney disease	31(20.9)	52(7.4)	<0.001	83(9.8)	5(2.5)	17(1.6)	0.408	22(1.8)	105(5.0)

Data are mean ± SD for continuous values or n (%) for category values

*TC* total cholesterol, *TG* triglyceride, *LDL-C* low density lipoprotein cholesterol

### Age and gender-specific prevalence of hyperuricemia and MCI of the study participants

The prevalence of hyperuricemia and MCI in this community elderly was 16.7% and 15.9% respectively. Prevalence of both two diseases increased linearly with increasing age group (*p* < 0.001). Women have a statistically higher prevalence of MCI than men (18.0% vs. 12.9%, *P* = 0.001). For hyperuricemia, men had a relatively higher prevalence in the elderly aged less than 70 yrs., but women had increased at a faster rate than men, however, the difference was not statistically significance (*p* > 0.05). The results were listed in Table 2.

**Table 2:** Prevalence of hyperuricemia and MCI by gender and age group

	Prevalence of hyperuricemia (%)				Prevalence of MCI (%)			
	Men	Female	*p*	Subtotal	Men	Female	*p*	Subtotal
Age group								
60–69 yrs	14.8	12.3	0.300	13.2	7.4	9.9	0.048	9.0
70–79 yrs	17.8	18.7	0.704	18.3	12.9	22.4	<0.001	18.5
≥ 80 yrs	22.6	24.1	0.815	23.2	26.1	38.0	0.039	30.9
p_trend_	0.036	0.002		<0.001	<0.001	<0.001		<0.001
subtotal	17.5	16.3	0.475	16.7	12.9	18.0	0.001	15.9

### Mean MMSE score and prevalence of MCI for participants with and without hyperuricemia

The mean MMSE score showed an increasing trend along with quarters of SUA levels among those without hyperuricemia in both genders (p for trend <0.05). The prevalence of MCI also showed the same trend, which decreased from 20.9% to 13.7% (*p* = 0.030). Among those participants who had hyperuricemia, the mean score of MMSE and the prevalence of MCI did not show statistically significant changing trend with SUA levels. The results were listed in Table 3. Also, we can see from Table 3, the mean score kept increasing for the first two quarters among those with hyperuricemia, and got decreased for the highest two quarters of SUA levels. The prevalence also decreased from 16.5% to 9.1% for the first three quarters, and changed to 13.6% for the highest quarter of SUA level.

**Table 3:** MMSE score and MCI Prevalence According to SUA quartiles by gender and hyperuricemia

	SUA quartiles by gender and hyperuricemia					
	Q_1_	Q_2_	Q_3_	Q_4_	*P*_*trend*_	total
Participants without hyperuricemia						
MMSE score						
Male	26.9 ± 3.9	27.0 ± 4.0	27.1 ± 3.4	27.2 ± 3.7	0.038	27.0 ± 3.8
Female	26.3 ± 3.8	26.5 ± 4.1	27.0 ± 3.9	27.1 ± 3.2	0.048	26.7 ± 3.8
Subtotal	26.6 ± 3.9	26.8 ± 4.1	27.0 ± 3.7	27.1 ± 3.4	0.031	26.8 ± 3.8
Prevalence of MCI						
Male	28(15.7)	23(13.7)	24(13.6)	21(11.9)	0.039	96(13.7)
Female	68(25.8)	52(20.1)	39(14.8)	36(13.6)	0.008	195(18.6)
Subtotal	96(20.9)	75(16.7)	63(15.2)	57(13.7)	0.030	291(16.6)
Participants with hyperuricemia						
MMSE score						
Male	27.8 ± 3.2	27.7 ± 2.5	27.7 ± 2.8	27.5 ± 2.2	0.965	27.7 ± 2.6
Female	26.7 ± 3.6	27.4 ± 2.7	27.2 ± 4.1	26.5 ± 3.4	0.549	27.0 ± 3.5
Subtotal	27.2 ± 3.4	27.5 ± 2.5	27.3 ± 3.6	27.0 ± 3.1	0.772	27.2 ± 3.1
Prevalence of MCI						
Male	5(13.5)	3(8.1)	4(10.8)	1(2.7)	0.155	13(8.8)
Female	9(18.8)	7(13.0)	4(7.8)	11(21.6)	0.854	31(15.2)
Subtotal	14(16.5)	10(11.0)	8(9.1)	12(13.6)	0.526	44(12.5)

### Multivariate associations of SUA level with MMSE score and MCI prevalence

Table 4 showed the multivariate associations between SUA and with MMSE score and MCI prevalence. This table showed that after adjusted age, gender, education, marital status, current smoking, current alcohol drinking, BMI, physical activity ≥ 0.5 h/day, family history of dementia, hypertension, cerebral vascular disease and diabetes, participants with higher SUA levels were at significantly elevated risk for MCI prevalence among those without hyperuricemia. For MMSE score, the β (95% CI) of SUA were 0.14(95% CI: 0.04–0.42) and 0.28(95% CI: 0.02–0.53) for men and women respectively. For MCI prevalence, the OR (95% CI) of quarters of SUA showed an increasing trend. The ORs were 1.01(95% CI: 0.69–1.48), 1.50(95% CI: 0.85–2.64), 1.65(95% CI: 1.12–2.43) and 1.53(95% CI: 1.00–2.33), 1.84(95% CI: 1.27–2.90), 1.92(95% CI: 1.02–3.35) for the second, third and highest quarters among men and women respectively (with the lowest quartile as the reference). Among the participants with hyperuricemia, the β (95% CI) for MMSE score were −0.03(95% CI: -0.56-0.39) and −0.01(95% CI: -0.43-0.37) for men and women. In the model of MCI prevalence, the ORs were also not statistically significant among participants with hyperuricemia.

**Table 4:** Multiple linear and logistic regression of SUA level for MMSE score and MCI prevalence

	SUA level (continuous)		SUA level (quartiles by gender)				
MMSE score	β(95% CI)	*P*	Q1	Q2 β(95% CI)	Q3 β(95% CI)	Q4 β(95% CI)	*P*_*trend*_
Participants with hyperuricemia							
Male	−0.03(−0.56–0.39)	0.721	1.00(Ref)	0.97(0.66–1.43)	0.92(0.58–1.26)	0.99(0.87–1.13)	0.544
Female	−0.01(−0.43–0.37)	0.882	1.00(Ref)	1.07(0.80–1.27)	1.05(0.78–1.31)	1.00(0.92–1.18)	0.922
Participants without hyperuricemia							
Male	0.14(0.04–0.42)	0.048	1.00(Ref)	1.01(0.69–1.48)	1.50(0.85–2.64)	1.65(1.12–2.43)	0.027
Female	0.28(0.02–0.53)	0.037	1.00(Ref)	1.53(1.00–2.33)	1.84(1.27–2.90)	1.92(1.02–3.35)	0.017
Prevalence of MCI	OR (95% CI)	*P*	Q1	Q2 OR (95% CI)	Q3 OR (95% CI)	Q4 OR (95% CI)	*P*_*trend*_
Participants with hyperuricemia							
Male	1.06(0.47–1.64)	0.126	1.00(Ref)	0.48(0.10–2.33)	0.54(0.12–2.40)	0.10(0.01–0.97)	0.256
Female	1.02(0.67–1.56)	0.915	1.00(Ref)	0.59(0.18–1.90)	0.32(0.08–1.23)	1.08(0.36–3.20)	0.246
Participants without hyperuricemia							
Male	0.82(0.68–0.99)	0.047	1.00(Ref)	0.95(0.50–1.78)	0.91(0.49–1.70)	0.72(0.38–1.36)	0.025
Female	0.80(0.65–0.97)	0.034	1.00(Ref)	0.87(0.45–1.70)	0.72(0.44–1.77)	0.64(0.39–1.06)	0.008

Adjusted for age, education, marital status, BMI, current smoking, current drinking, physical activity ≥ 0.5 h/day, family history of dementia, hypertension, cerebral vascular disease and diabetes

We also ascertained the association of SUA level with MMSE score and MCI prevalence in the sensitivity analysis and the results were similar (Additional file 1: Appendix Table S1 and S2). When participants with cerebral vascular disease (*n* = 267, 12.7%) and kidney disease (*n* = 105, 5.0%) were excluded, the βs and ORs were similar with that in the Table 4. Higher SUA level was related with better cognitive function when SUA was in the normal range. There was also still no significant association among participants with hyperuricemia.

## Discussion

In this representative community elderly based study; we evaluated the association of SUA level and cognitive function. The results that higher SUA level within normal range were positively associated with a decreased prevalence of MCI even after adjusting for demographic and other potential confounders. However, this association was not significant among participants with hyperuricemia.

Different from the high risk and harm of SUA level and cardiovascular diseases [25], a number of studies have provided evidence that SUA had protective effect on cognitive function and is beneficial to reduce dementia [14–16]. In this representative sample of Chinese urban elderly, we also found a graded positive association between hyperuricemia and cognitive function. When in the normal range, higher SUA level was associated with higher MMSE score and lower risk of MCI prevalence. And cognitive function was found to rise along with the increase of quarters of SUA levels. This is in accordance with previous studies. Molshatzki found that after 10 years follow-up, low uric acid levels in patients with preexisting cardiovascular disease are associated with poorer cognitive function [14]. Méndez-Hernández revealed that higher levels of uric acid are associated with a decreased risk of dementia using a case-control study in a Mexican population [13]. Yili Wu found that among Chinese aged 50–74 years, prevalence of cognitive disorder declined across UA tertiles [9]. The mechanism between higher SUA level and better cognitive function was not clear; one possible explanation was oxidative stress. SUA is a water-soluble antioxidant, which accounts for more than half of the free radical scavenging activity [17]. SUA could exert neuro-protective effects against Alzheimer’s disease via its antioxidant capacities [16].

The present study also provides evidence that elevated SUA level was not associated with MCI among those participants with hyperuricemia. When SUA level was higher than the normal range, there was no statistically significant positive correlation with cognitive function. This result confirmed previous studies. A study about elderly women from U.S. showed higher baseline SUA levels was associated with poorer cognitive performance (*p* = 0.048). Another study from Italy showed that participants who had the highest SUA tertile had higher risk (OR = 3.32, 95%CI: 1.06–10.42) to suffer from dementia based on 1016 community-dwellings. On the other hand, there were several studies revealed that higher SUA levels were related to higher prevalence of gout and cardiovascular diseases [26, 27]. So the key question is what is the appreciate cut point for SUA levels. There is urgent need for long-time follow-up cohort studies to verify the optimal diagnostic boundary value, which would maximize the benefits, not only for gout and cardiovascular diseases, but also for MCI and dementia.

The study had several potential limitations. First, the SUA level was measured once, and may not reflect the true value, and did not consider the fluctuation. Second, the limitations of cross-sectional study lowered strength of evidence for causal inference. Long time follow up study are needed to confirm the results. Third, results among hyperuricemia individuals are based on very few cases of MCI patients, thus results might not be reliable. Fourth, there were several possible confounding factors we didn’t consider ApoE genotype and drug treatment status. On the other hand, the study also some advantages. We used strict training process and quality assurance programs during the whole study. The selected community was representative metropolitan area of the geographic and economic characteristics in Beijing, and the response rate was high. So this population-based study with large sample and high response rate would have a good chance to better analysis the association between SUA level and cognitive function. Second, the SUA level in this study was carefully analyzed using the normal range and higher SUA level in hyperuricemia, which is different from most previous studies. The results also showed that it’s not the simple “the higher the better” or “no correlation” or “the lower the better”. The association results were different with SUA levels.

## Conclusion

In summary, the present study showed higher SUA level are related to lower prevalence of MCI and better cognitive function, only when SUA level was in the normal range among Chinese community elderly population. This indicated that further studies should focus on the appropriate SUA level, which is helpful to reduce both dementia and cardiovascular diseases.

## Additional file

Additional file 1: Appendix Tables (appendix Table S1 and S2). Multiple linear and logistic regression of SUA level for MMSE score and MCI prevalence for participants without cerebral vascular disease or without kidney diseases**.** We ascertained the association of SUA level with MMSE score and MCI prevalence in the sensitivity analysis (Appendix Table S1 and S2). When participants with cerebral vascular disease (*n* = 267, 12.7%) and kidney disease (*n* = 105, 5.0%) were excluded, the βs and ORs were similar with that in the Table 4 (all participants). (DOC 62 kb)
