# Supplementary material for: Relationship between serum uric acid level and mild cognitive impairment in Chinese community elderly
Source: BMC Neurol. 2017 Aug 1;17:146. doi: 10.1186/s12883-017-0929-8 (PMC5539640; doi:10.1186/s12883-017-0929-8)
Supplement: Additional file 1: — Appendix Tables (appendix Table S1 and S2). Multiple linear and logistic regression of SUA level for MMSE score and MCI prevalence for participants without cerebral vascular disease or without kidney diseases. We ascertained the association of SUA level with MMSE score and MCI prevalence in the sensitivity analysis (Appendix Table S1 and S2). When participants with cerebral vascular disease (n = 267, 12.7%) and kidney disease (n = 105, 5.0%) were excluded, the βs and ORs were similar with that in the Table 4 (all participants). (DOC 62 kb) [file 12883_2017_929_MOESM1_ESM.doc]

**Additional file 1: Appendix Table S1.** Multiple linear and logistic regression of SUA level for MMSE score and MCI prevalence for participants without cerebral vascular disease (n=1835)

|  | SUA level (continuous) | |  | SUA level (quartiles by gender) | | | | |
| --- | --- | --- | --- | --- | --- | --- | --- | --- |
| MMSE score | β(95%CI) | *P* |  | Q1 | Q2 β(95%CI) | Q3 β(95%CI) | Q4 β(95%CI) | *P_trend_* |
| Participants with hyperuricemia |  |  |  |  |  |  |  |  |
| Male | -0.03(-0.54-0.40) | 0.758 |  | 1.00(Ref) | 0.96(0.61-1.48) | 0.93(0.50-1.30) | 1.00(0.88-1.19) | 0.496 |
| Female | -0.01(-0.46-0.41) | 0.814 |  | 1.00(Ref) | 1.06(0.81-1.28) | 1.05(0.74-1.36) | 1.02(0.89-1.32) | 0.894 |
| Participants without hyperuricemia |  |  |  |  |  |  |  |  |
| Male | 0.14(0.03-0.45) | 0.047 |  | 1.00(Ref) | 1.01(0.69-1.48) | 1.50(0.85-2.64) | 1.64(1.11-2.42) | 0.029 |
| Female | 0.29(0.03-0.56) | 0.038 |  | 1.00(Ref) | 1.52(0.98-2.30) | 1.80(1.20-2.90) | 1.93(1.02-3.38) | 0.018 |
| Prevalence of MCI | OR (95%CI) | *P* |  | Q1 | Q2 OR (95%CI) | Q3 OR (95%CI) | Q4 OR (95%CI) | *P_trend_* |
| Participants with hyperuricemia |  |  |  |  |  |  |  |  |
| Male | 1.06(0.45-1.67) | 0.132 |  | 1.00(Ref) | 0.48(0.10-2.33) | 0.56(0.10-2.52) | 0.12(0.01-0.88) | 0.262 |
| Female | 1.02(0.63-1.62) | 0.924 |  | 1.00(Ref) | 0.59(0.16-1.93) | 0.31(0.07-1.28) | 1.08(0.33-3.26) | 0.251 |
| Participants without hyperuricemia |  |  |  |  |  |  |  |  |
| Male | 0.80(0.61-0.99) | 0.048 |  | 1.00(Ref) | 0.95(0.48-1.80) | 0.88(0.41-1.81) | 0.70(0.35-1.45) | 0.025 |
| Female | 0.78(0.60-0.98) | 0.036 |  | 1.00(Ref) | 0.85(0.43-1.80) | 0.70(0.40-1.85) | 0.59(0.30-1.16) | 0.010 |

*Adjusted for age, education, marital status, BMI, current smoking, current drinking, physical activity≥0.5h/day, family history of dementia, hypertension, cerebral vascular disease and diabetes.*

**Additional file 1: Appendix Table S2.** Multiple linear and logistic regression of SUA level for MMSE score and MCI prevalence for participants without kidney diseases (n=1997)

|  | SUA level (continuous) | |  | SUA level (quartiles by gender) | | | | |
| --- | --- | --- | --- | --- | --- | --- | --- | --- |
| MMSE score | β(95%CI) | *P* |  | Q1 | Q2 β(95%CI) | Q3 β(95%CI) | Q4 β(95%CI) | *P_trend_* |
| Participants with hyperuricemia |  |  |  |  |  |  |  |  |
| Male | -0.03(-0.51-0.39) | 0.786 |  | 1.00(Ref) | 0.97(0.60-1.46) | 0.94(0.50-1.31) | 1.00(0.88-1.21) | 0.532 |
| Female | -0.01(-0.44-0.40) | 0.802 |  | 1.00(Ref) | 1.06(0.80-1.26) | 1.05(0.76-1.32) | 1.02(0.90-1.34) | 0.874 |
| Participants without hyperuricemia |  |  |  |  |  |  |  |  |
| Male | 0.15(0.03-0.41) | 0.048 |  | 1.00(Ref) | 1.01(0.69-1.47) | 1.51(0.86-2.62) | 1.60(1.19-2.29) | 0.033 |
| Female | 0.28(0.03-0.54) | 0.039 |  | 1.00(Ref) | 1.52(0.98-2.32) | 1.76(1.20-2.52) | 1.91(1.03-3.21) | 0.020 |
| Prevalence of MCI | OR (95%CI) | *P* |  | Q1 | Q2 OR (95%CI) | Q3 OR (95%CI) | Q4 OR (95%CI) | *P_trend_* |
| Participants with hyperuricemia |  |  |  |  |  |  |  |  |
| Male | 1.06(0.43-1.61) | 0.128 |  | 1.00(Ref) | 0.47(0.11-2.32) | 0.58(0.11-2.56) | 0.12(0.01-0.86) | 0.274 |
| Female | 1.02(0.66-1.58) | 0.903 |  | 1.00(Ref) | 0.58(0.17-1.85) | 0.32(0.08-1.26) | 1.07(0.36-3.30) | 0.261 |
| Participants without hyperuricemia |  |  |  |  |  |  |  |  |
| Male | 0.78(0.59-0.98) | 0.047 |  | 1.00(Ref) | 0.96(0.38-1.81) | 0.85(0.40-1.69) | 0.72(0.41-1.38) | 0.027 |
| Female | 0.78(0.60-0.97) | 0.038 |  | 1.00(Ref) | 0.88(0.47-1.82) | 0.71(0.38-1.90) | 0.61(0.29-1.16) | 0.014 |

*Adjusted for age, education, marital status, BMI, current smoking, current drinking, physical activity≥0.5h/day, family history of dementia, hypertension, cerebral vascular disease and diabetes.*
